# Supplementary material for: Hybrid Models and Biological Model Reduction with PyDSTool
Source: PLoS Comput Biol. 2012 Aug 9;8(8):e1002628. doi: 10.1371/journal.pcbi.1002628 (PMC3415397; doi:10.1371/journal.pcbi.1002628)
Supplement: Text S4 — Complete source code for the PyDSTool package (version 0.88.120504). Includes API documentation and help files linking to web pages. This file is identical to the current public release on Sourceforge.net. (ZIP) [file pcbi.1002628.s004.zip › PyDSTool/html/PyDSTool.Toolbox.optimizers.step.partial_step.PartialStep-class.html]

xml version="1.0" encoding="ascii"?


PyDSTool.Toolbox.optimizers.step.partial\_step.PartialStep


| Home | Trees | Indices | Help | PyDSTool |
| --- | --- | --- | --- | --- |

Package PyDSTool ::
Package Toolbox ::
Package optimizers ::
Package step ::
Module partial\_step ::
Class PartialStep

[hide private]

[frames] | no frames]

# Class PartialStep

source code

```
object --+
         |
        PartialStep
```

---

A partial step


|  |  |
| --- | --- |
| Instance Methods | [hide private] |

|  |  |
| --- | --- |
| \_\_init\_\_(self, step, nb\_chunks, indice=None)  Allows only a part of the set of parameters to be updated | source code |

|  |  |
| --- | --- |
| \_\_call\_\_(self, function, point, state)  Computes a step based on a function and a point | source code |

**Inherited from `object`**:
`__delattr__`,
`__getattribute__`,
`__hash__`,
`__new__`,
`__reduce__`,
`__reduce_ex__`,
`__repr__`,
`__setattr__`,
`__str__`


|  |  |
| --- | --- |
| Properties | [hide private] |

**Inherited from `object`**:
`__class__`


|  |  |
| --- | --- |
| Method Details | [hide private] |

|  |  |
| --- | --- |
| \_\_init\_\_(self, step, nb\_chunks, indice=None)  *(Constructor)* | source code |

Allows only a part of the set of parameters to be updated

- step is the step that will be decorated
- nb\_chunks is the number of chunks in the parameter set
- indice is the chunk to update each time. if None, a random indice is
  drawn each time

Overrides: object.\_\_init\_\_

  


| Home | Trees | Indices | Help | PyDSTool |
| --- | --- | --- | --- | --- |

|  |  |
| --- | --- |
| Generated by Epydoc 3.0.1 on Fri May 4 15:24:09 2012 | http://epydoc.sourceforge.net |
